# Long noncoding RNA NKILA transferred by astrocyte-derived extracellular vesicles protects against neuronal injury by upregulating NLRX1 through binding to mir-195 in traumatic brain injury

**DOI:** 10.18632/aging.202618

**Published:** 2021-03-03

**Authors:** Bin He, Wei Chen, Jingsong Zeng, Wusong Tong, Ping Zheng

**Affiliations:** 1 Department of Neurosurgery, Shanghai University of Medicine and Health Sciences, Shanghai 201318, P.R. China; 2 Department of Neurosurgery, Shanghai Pudong New Area People’s Hospital, Shanghai 201200, P.R. China

**Keywords:** traumatic brain injury, long noncoding RNA NKILA, microRNA-195, NLRX1, extracellular vesicles

## Abstract

The study aims to investigate the effects of long noncoding RNA (lncRNA) transmitted nuclear factor-κB interacting lncRNA (NKILA)-containing astrocyte-derived small extracellular vesicles (EVs) on traumatic brain injury (TBI). TBI was modeled *in vitro* by exposing human neurons to mechanical injury and *in vivo* by controlled cortical impact in a mouse model. The gain- and loss-function approaches were conducted in injured neurons to explore the role of NKILA, microRNA-195 (miR-195) and nucleotide-binding leucine-rich repeat containing family member X1 (NLRX1) in neuronal injury. EVs extracted from NKILA-overexpressing astrocytes were used to treat injured neurons. It was revealed that NKILA was downregulated in injured neurons. Astrocyte co-culture participated in the upregulation of NKILA in injured neurons. Additionally, NKILA could competitively bind to miR-195 that directly targeted NLRX1. Next, the upregulation of NLRX1 or NKILA relived neuronal injury by promoting neuronal proliferation but inhibiting apoptosis. Astrocyte-derived EVs transferred NKILA into neurons, which led to the downregulation of miR-195, upregulation of NLRX1, increased cell proliferation, and decreased cell apoptosis. The *in vivo* experiments validated that NKILA-containing EVs promoted brain recovery following TBI. Collectively, astrocyte-derived EVs carrying NKILA was found to alleviate neuronal injury in TBI by competitively binding to miR-195 and upregulating NLRX1.

## INTRODUCTION

As a public health problem around the globe, traumatic brain injury (TBI) refers to the dysregulation of brain function or other evidence of external force-induced brain pathology [1]. The secondary biochemical changes will occur after occurrence of TBI, which subsequently contributes to tissue damage and associated with neuronal cell death [2]. At present, medical and surgical therapies were commonly used for the treatment of TBI to minimize secondary brain injury [3]. As the special glial cells, astrocytes are almost five times more in number than neurons and adjacent to the whole central nervous system (CNS), which play complex functions in healthy CNS [4]. Evidence exists reporting that astrocytes serve as housekeepers of the CNS and are pivotal for CNS development, homeostasis and defense [5]. A prior study discovered astrocytes as the critical regulator of cortical spreading depression and TBI [6]. Of interest, extracellular vesicles (EVs) can be constitutively released by astrocytes [7].

EVs consisting of microvesicles, exosomes, and apoptotic bodies have been implicated as cargo-carrying vesicles regulating communication among various cells and tissues, including the CNS [8]. It has been demonstrated that EVs secreted from astrocytes could be conduits for transferring the cargo of macromolecules to neighboring as well as distant cells, leading to a wide range of functional changes in the recipient cells [9]. Astrocyte-derived EVs have also been proposed to confer neuroreparative functions by regulating neuronal uptake, differentiation and firing [10, 11]. EVs have been deciphered to participate in cellular communication by shuttling long noncoding RNAs (lncRNAs), microRNAs (miRNAs), proteins, and mRNAs [12].

It should be noted that a variety of lncRNAs are reported to be aberrantly expressed in hippocampus of rats with TBI [13]. Accumulating evidences have showed that nuclear transcription factor NF-κB interacting lncRNA (NKILA) can inhibit the activation of the NF-κB signaling pathway where the activation was associated with inflammatory response following TBI [14, 15]. Moreover, it has been documented that lncRNAs might act as ceRNAs so as to modulate the expression of different miRNAs in a cell-type dependent manner [16]. Interestingly, NKILA was predicted to present putative binding sites with miR-195 in the RNA22 website in the present study. As a member of the miR-15/16/195/424/497 family, miR-195 was activated in many diseases, including schizophrenia, heart failure and cancers [17]. Increased miR-195 expression suppresses autophagy to promote neuropathic pain following peripheral nerve injury [18]. Intriguingly, Nod-like receptor (NLR) X1 was a target gene of miR-195 [19]. As a mitochondrial NOD-like receptor, NLRX1 promotes the production of reactive oxygen species to amplify NF-κB and JNK signaling pathways [20]. A prior study revealed that NLRX1 exerted a suppressive effect on TBI *via* negative regulation of the NF-κB signaling pathway [21]. Based on the discussion above, a hypothesis was proposed that EV-encapsulated NKILA might participate in the development of TBI *via* miR-195-regulated NLRX1. Therefore, the present study was designed to investigate the effect of astrocyte-derived EVs carrying NKILA on neuronal injury following TBI *via* NLRX1 with a cell injury model of human neurons and a mouse model of TBI.

## RESULTS

### Astrocytes promote the recovery of injured neurons by inducing NKILA expression

**Figure 1:**
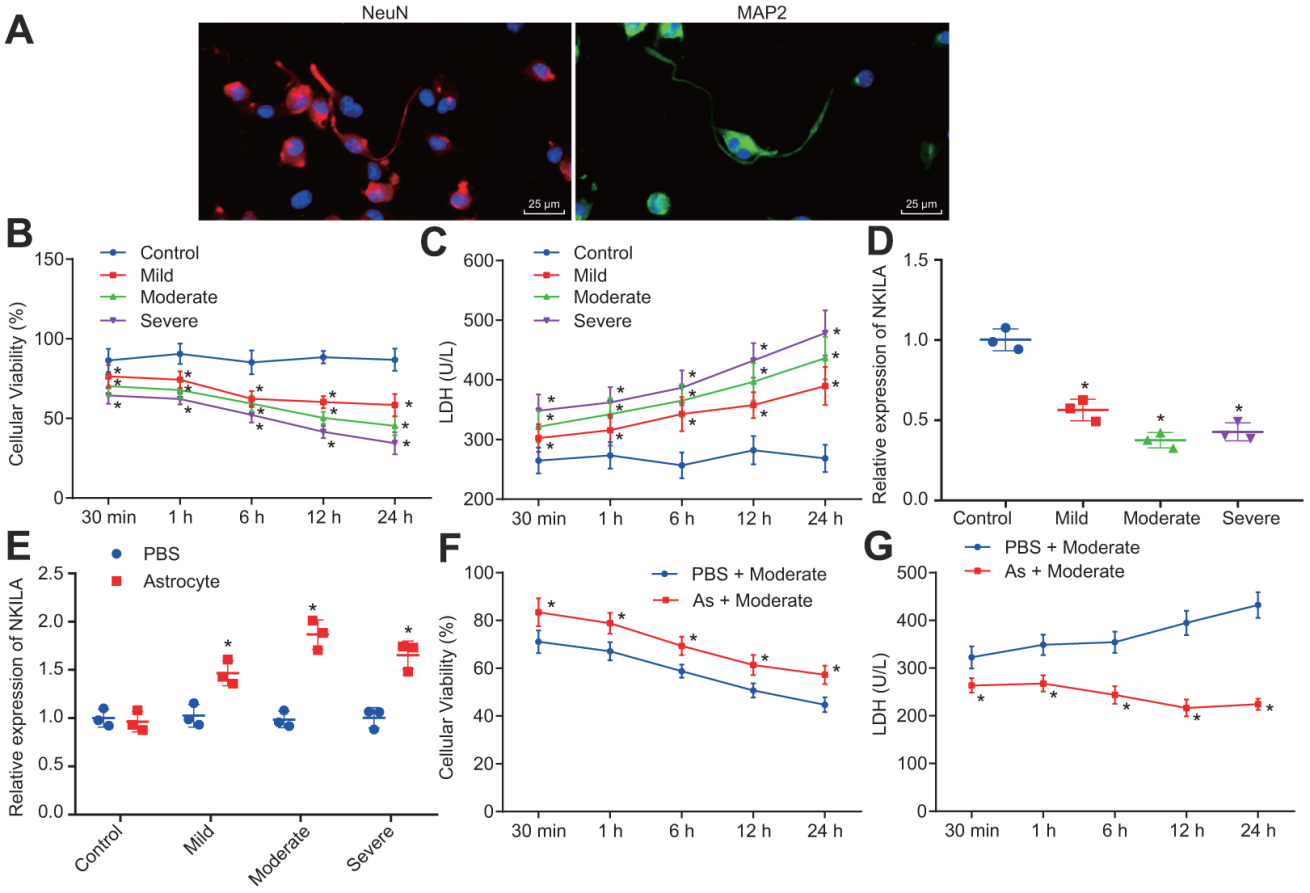
**Astrocytes upregulates NKILA to promote recovery of injured neurons**. (**A**) immunochemical staining of MAP2 and NeuN expression in neurons (× 400). (**B**) proliferation of neurons with mild, moderate or severe injury detected by CCK-8 assay. (**C**) LDH content in the culture medium of neurons with mild, moderate or severe injury. (**D**) NKILA expression in neurons with mild, moderate or severe injury detected by RT-qPCR. (**E**) NKILA expression in injured neurons after co-culture of astrocytes detected by RT-qPCR. (**F**) neuronal proliferation in injured neurons after co-culture of astrocytes detected by CCK-8 assay. (**G**) LDH content in injured neuron after co-culture of astrocytes. All data were measurement data and expressed as mean ± standard deviation. Independent sample *t* test was used for comparison between two groups (**E**). The one-way ANOVA was used for comparison among multiple groups, followed by Tukey’s post-hoc test (**D**) and two-way ANOVA for comparisons between time-based measurements within each group (**B**, **C**, **F**, **G**). All experiments were done at least three independent times. * *p* < 0.05 compared with control neurons or moderately injured neurons cultured with PBS.

Astrocytes have been previously reported to secrete a variety of substances to protect the CNS [22]. To characterize the protective effect of astrocytes on neurons, a mechanical injury model of human neurons was constructed *in vitro*. Initially, we identified the human neurons with immunofluorescence, revealing that the neurons expressed microtubule-associated protein 2 (MAP2) and neuron-specific protein (NeuN) (Figure 1A). Thereafter, the proliferation in neurons were strikingly decreased in mild, moderate or severe injury, shortly after 30 min injury, peaking at 24 h, and cell viability decreased with the severity of injury (*p* < 0.05; Figure 1B). In addition, lactate dehydrogenase (LDH) content was significantly higher in injured neurons compared to control neurons 30 min post-injury (*p* < 0.05; Figure 1C). Moreover, the reverse transcription-quantitative polymerase chain reaction (RT-qPCR) results confirmed that NKILA expression was downregulated in injured neurons (Figure 1D), while the co-culture of astrocytes upregulated NKILA expression in injured neurons (Figure 1E). Meanwhile, when compared to the injured neurons culture, the cell viability was increased (Figure 1F) while LDH content (Figure 1G) was decreased in injured neurons co-cultured with astrocytes (*p* < 0.05). These results demonstrate that the stimulated NKILA expression accelerates the recovery of injured neurons.

### NKILA overexpression promotes proliferation and inhibits apoptosis in injured neurons

**Figure 2:**
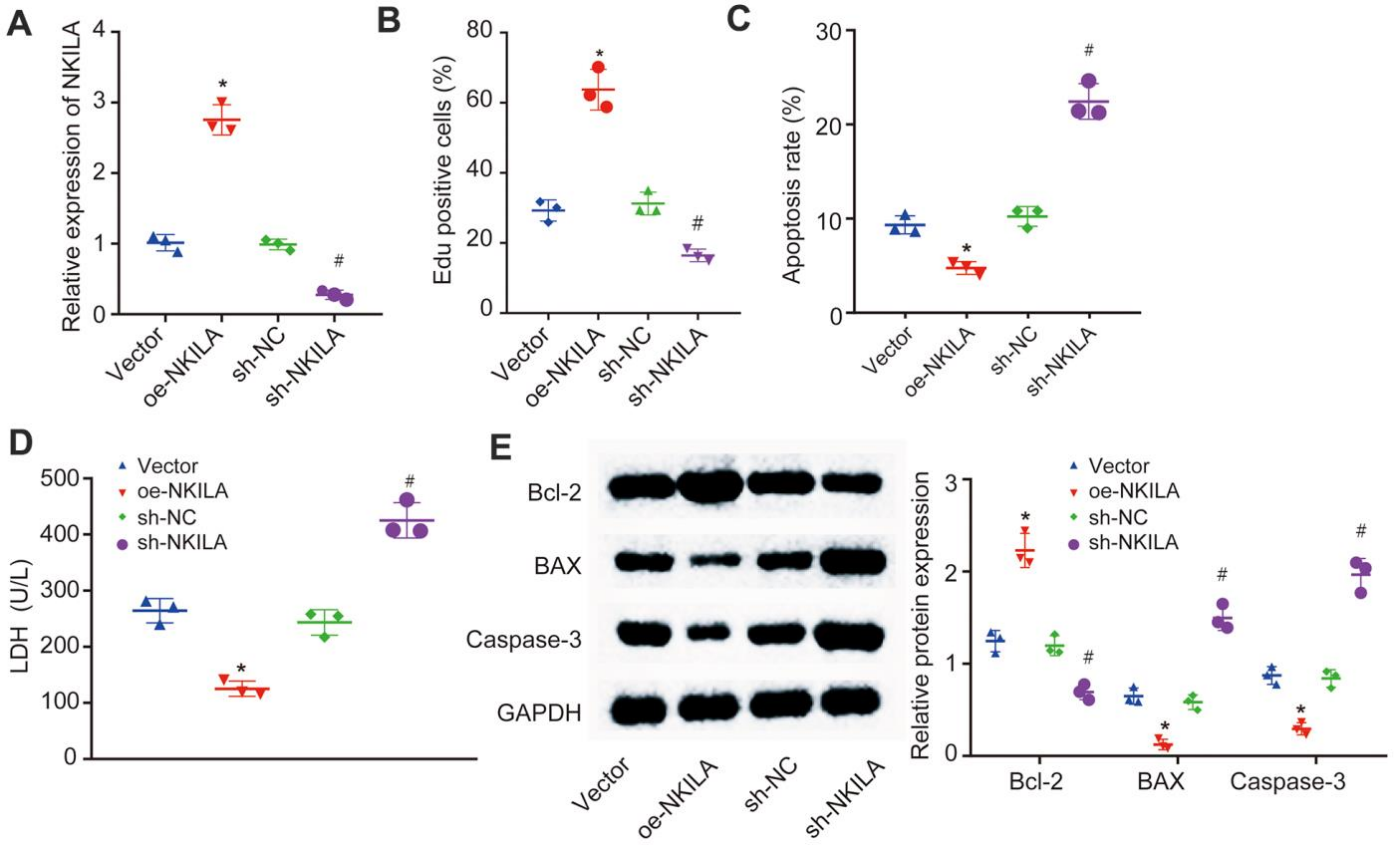
**NKILA promotes proliferation and inhibits apoptosis of injured neurons**. The injured neurons were introduced with oe-NKILA, sh-NKILA, vector or sh-NC. (**A**) NKILA expression in injured neurons after treatment measured by RT-qPCR. (**B**) cell proliferation of injured neurons after treatment measured by EdU assay. (**C**) quantitative analysis for cell apoptosis of injured neurons after treatment measured by flow cytometry. (**D**) LDH content in injured neurons after treatment. (**E**) protein expression of apoptosis-related factors (Bcl-2, Bax and Caspase-3) in injured neurons after treatment measured by Western blot analysis. * *p* < 0.05 compared with neurons treated with vector; # *p* < 0.05 compared with neurons treated with sh-NC. All data were measurement data and expressed as mean ± standard deviation. One-way ANOVA was used for comparison among multiple groups, followed by Tukey’s post-hoc test. The cell experiment was repeated three times.

To further characterize the recovery effect of NKILA overexpression in injured neurons, NKILA were upregulated and silenced in injured neurons (Figure 2A). Then, EdU assay, propidium iodide (PI) and Annexin labeling and quantification were conducted to measure the neuronal proliferation and apoptosis. When compared with neurons treated with vector, an increase in the proliferation and LDH content and a reduction in apoptosis were observed in neurons overexpressing NKILA (*p* < 0.05; Figure 2B–2D). Meanwhile, Western blot analysis revealed that the expression of anti-apoptotic gene (Bcl-2) was significantly higher, while the expression of pro-apoptotic genes [Bcl-2-Associated X (Bax) and Caspase-3] was significantly lower in overexpressing (oe) NKILA-treated neurons when compared with vector-treated neurons (*p* < 0.05; Figure 2E). However, these results were completely opposite in short hairpin (sh)-NKILA-treated neurons versus sh-NC-treated neurons. Taken together, the upregulation of NKILA promotes the functional recovery of injured neurons.

### NKILA competitively binds to miR-195

**Figure 3:**
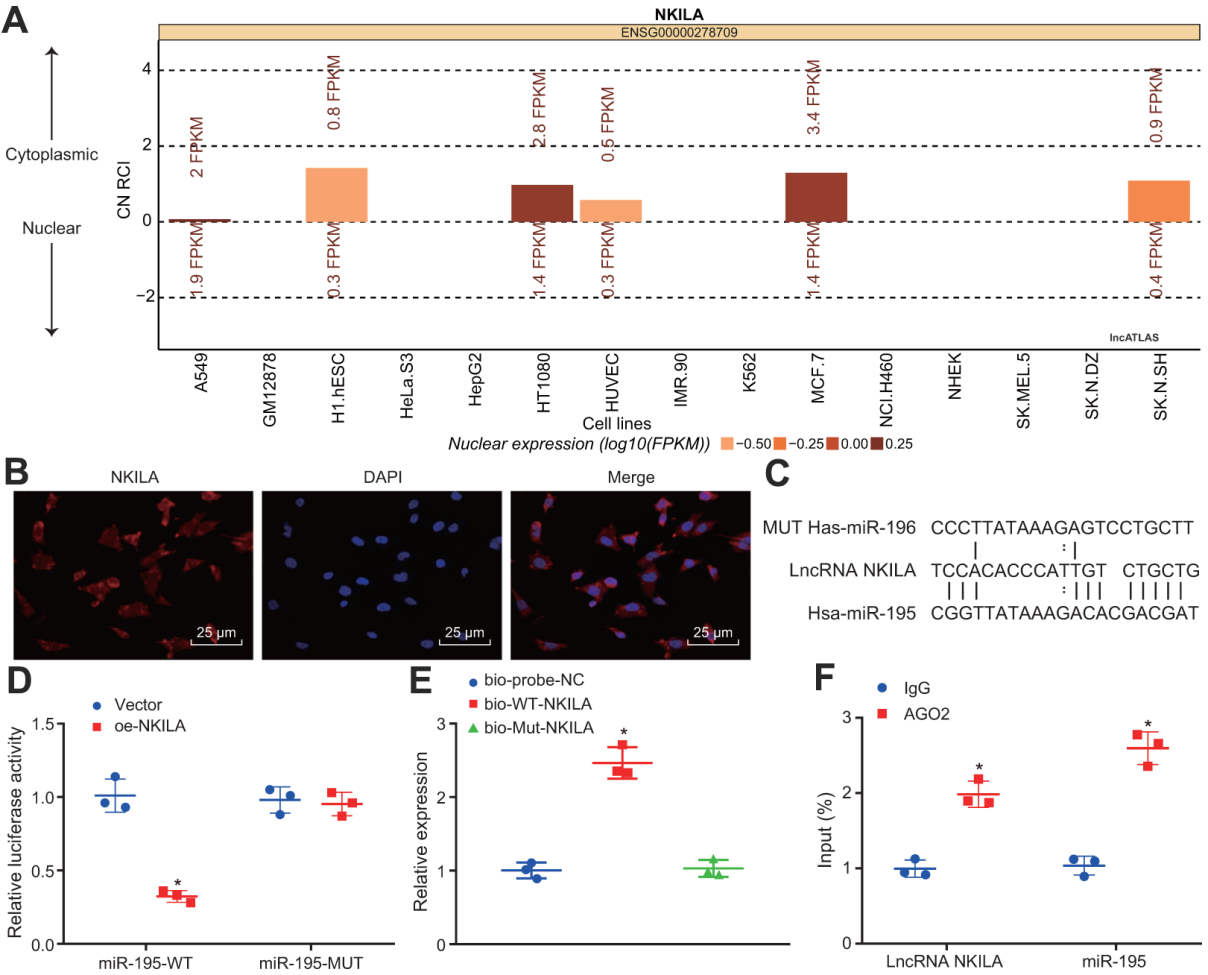
**NKILA competitively binds to miR-195**. (**A**) the subcellular location of NKILA in neurons predicted by the lncATLAS website. (**B**) the subcellular localization of NKILA in neurons detected by FISH assay (× 400). (**C**) the potential binding sites between NKILA and miR-195 predicted by RNA22. (**D**) the binding relationship between NKILA and miR-195 measured by dual-luciferase reporter gene assay. * *p* < 0.05 compared with the treatment of vector. (**E**) the enrichment of miR-195 caused by NKILA detected by RNA pull-down. * *p* < 0.05 compared with the treatment of Bio-probe NC. (**F**) the binding of NKILA and miR-195 with AGO2 determined by RIP assay. * *p* < 0.05 compared with IgG. All data were measurement data and expressed as mean ± standard deviation. Unpaired *t* test was used for pairwise comparison in panel D and F, and one-way ANOVA followed by Tukey’s post-hoc test was used for comparison in panel E. The cell experiment was repeated three times.

The NKILA subcellular location was predicted using the lncATLAS website, which demonstrated that NKILA was located in the cytoplasm (Figure 3A). Then, the result of fluorescence *in situ* hybridization (FISH) assay further substantiated that NKILA was located in the cytoplasm of neurons (Figure 3B). Meanwhile, the RNA22 website predicted binding sites between NKILA and miR-195 (Figure 3C). Dual-luciferase reporter gene assay further validated that when compared with the treatment of vector, the luciferase activity of miR-195-wild type (WT) was inhibited by the treatment of oe-NKILA (*p* < 0.05), while no obvious change of luciferase activity of miR-195-mutated (MUT) was observed (*p* > 0.05; Figure 3D), suggesting that NKILA could competitively bind to miR-195. Additionally, RNA pull-down displayed that compared with the treatment of Bio-probe NC, the enrichment of miR-195 remained unchanged following the treatment of Bio-MUT-NKILA (*p* > 0.05), but was significantly increased after the treatment of Bio-WT-NKILA (*p* < 0.05; Figure 3E), indicating that Bio-Wt-NKILA could promote the enrichment of miR-195. As depicted in Figure 3F, when compared with immunoglobulin G (IgG), the binding of NKILA and miR-195 to AGO2 were increased significantly (*p* < 0.05), suggesting that NKILA could bind to AGO2 protein and miR-195. Based on the aforementioned results, NKILA competitively binds to miR-195.

### NKILA upregulates NLRX1 by competitively binding to miR-195

**Figure 4:**
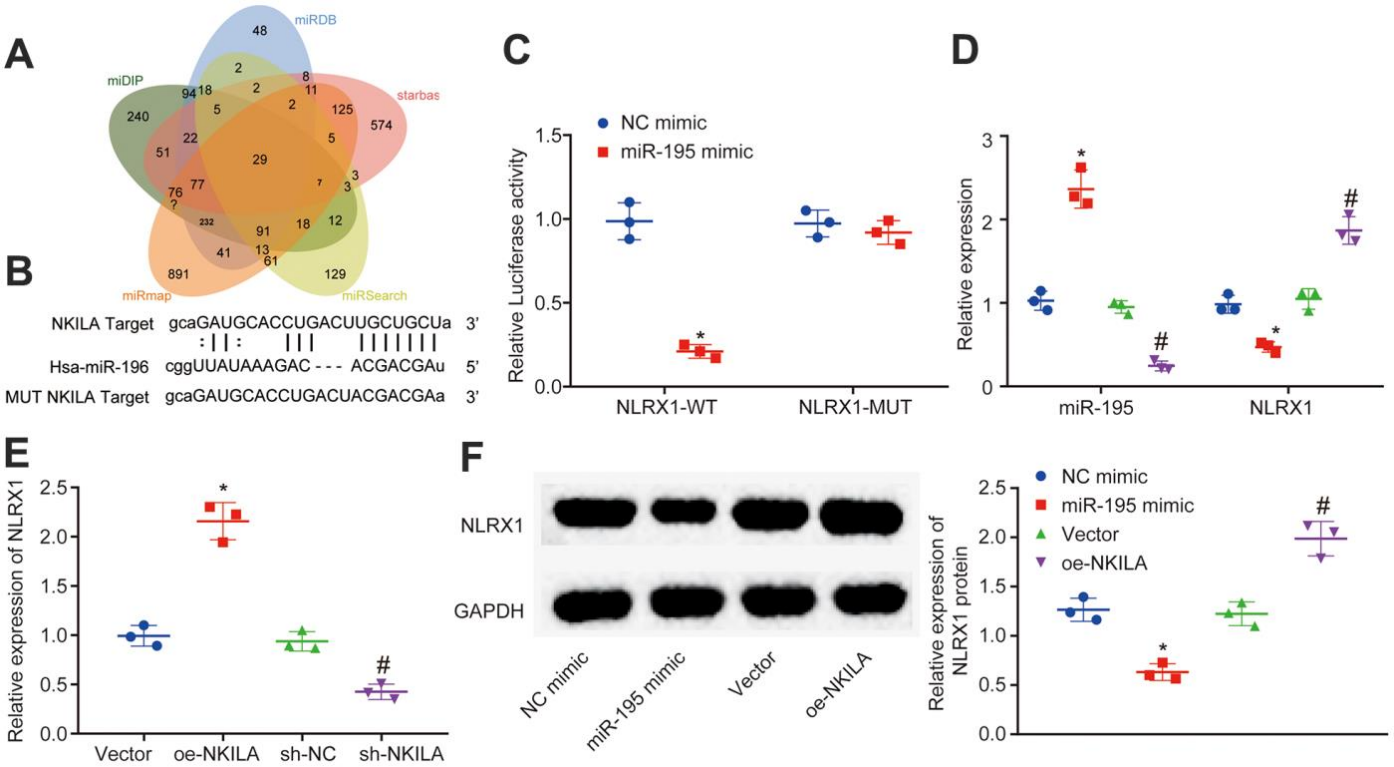
**NKILA acts as ceRNA of miR-195 to upregulate NLRX1**. (**A**) Venn analysis of target genes of miR-195 obtained from miDIP, miRDB and starbase. (**B**) putative binding sites between NLRX1 and miR-195 predicted in RNA22. (**C**) the binding relationship between NLRX1 and miR-195 verified by dual-luciferase reporter gene assay. * *p* < 0.05 compared with the treatment of NC mimic. (**D**) NLRX1 and miR-195 expression in neurons after alteration of miR-195 detected by RT-qPCR. * *p* < 0.05 compared with the treatment of NC mimic. # *p* < 0.05 compared with the treatment of NC inhibitor. (**E**) NLRX1 mRNA expression in neurons after alteration of NKILA detected by RT-qPCR. * *p* < 0.05 compared with the treatment of vector. # *p* < 0.05 compared with the treatment of sh-NC. (**F**) NLRX1 protein expression in neurons after overexpression of NKILA or miR-195 measured by Western blot analysis. * *p* < 0.05 compared with the treatment of NC mimic. # *p* < 0.05 compared with the treatment of vector. All data were measurement data and expressed as mean ± standard deviation. Unpaired *t* test was used for comparison between two groups. The one-way ANOVA was used for comparison among multiple groups, followed by Tukey’s post-hoc test. The cell experiment was repeated three times.

To identify the downstream mechanism of NKILA, the predicted target genes of miR-195 in miDIP, miRDB and starbase were intersected, where 29 intersected target genes were obtained (Figure 4A). Among these intersected genes, the loss of NLRX1 has been reported to aggravate the neurological injury and NF-κB signal after brain injury [23]. However, the mechanism of NLRX1 expression regulated by NKILA-mediated miR-195 to protect neurons from TBI remains undiscovered. Therefore, NLRX1 was selected as a candidate target gene. Based on RNA22 website, there were putative binding sites between NLRX1 and miR-195 (Figure 4B). Moreover, dual-luciferase reporter gene assay demonstrated that when compared with the treatment of NC mimic, the luciferase activity of NLRX1-3'UTR-WT was decreased by the treatment of miR-195 mimic (*p* < 0.05), while no obvious change of luciferase activity was observed in NLRX1-3'UTR-MUT (*p* > 0.05; Figure 4C), suggesting that miR-195 could target and negatively regulate NLRX1. The result of RT-qPCR revealed that the treatment of miR-195 mimic increased the miR-195 expression and decreased the NLRX1 expression, while the treatment of miR-195 inhibitor induced the opposite trends (Figure 4D). Meanwhile, RT-qPCR also demonstrated that NLRX1 expression was significantly increased after NKILA overexpression, but significantly decreased following NKILA inhibition (Figure 4E). Western blot analysis was performed to determine the NLRX1 protein expression. The results revealed that the protein expression after treatment of miR-195 mimic in NLRX1 was decreased, but increased in NLRX1 protein expression after treatment of oe-NKILA (Figure 4F). These results demonstrated that NKILA increases NLRX1 expression by binding to miR-195.

### Overexpression of NKILA induces proliferation and suppresses apoptosis in injured neurons *via* miR-195-mediated upregulation of NLRX1

**Figure 5:**
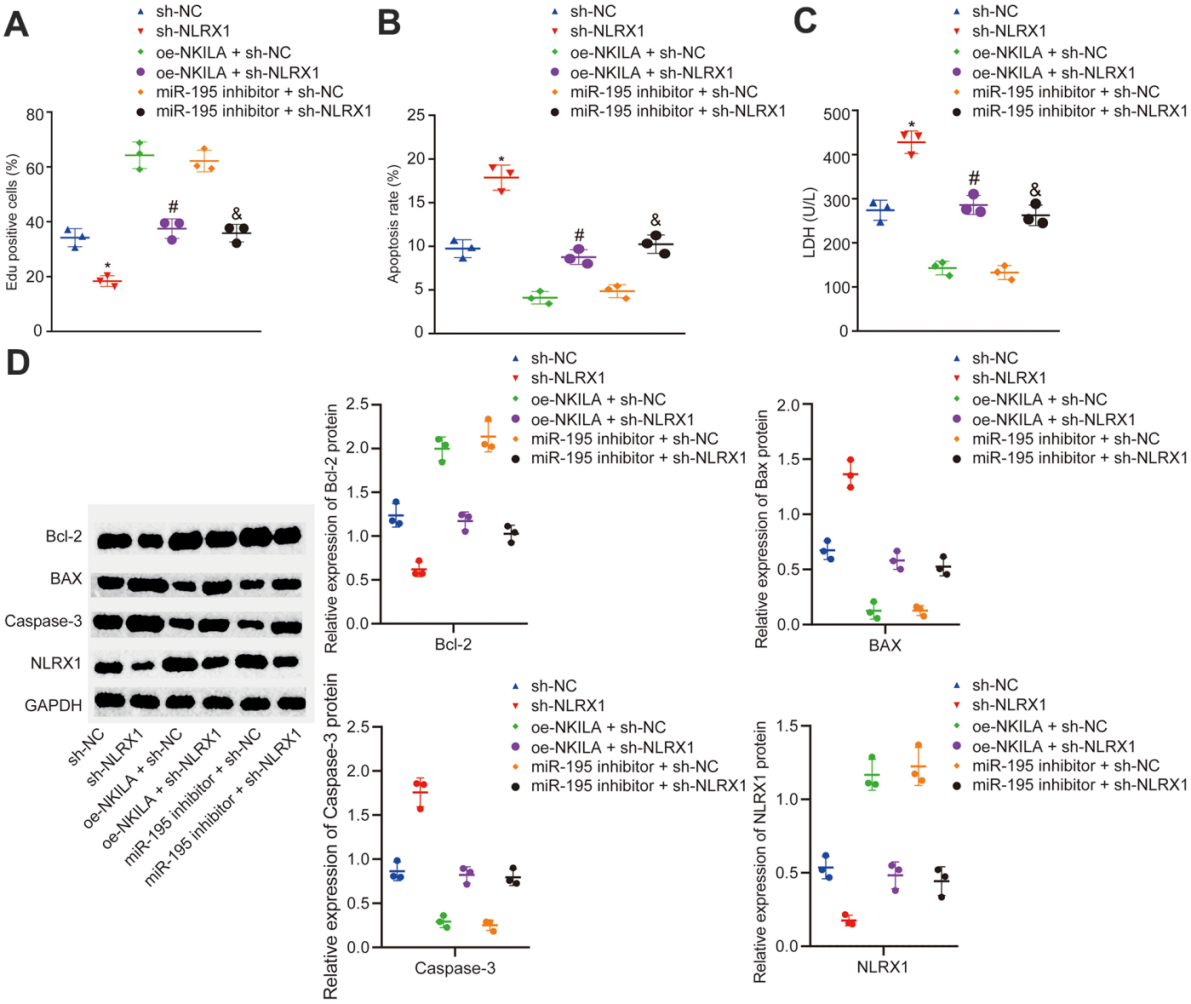
**NKILA promotes proliferation and inhibits apoptosis of injured neurons by increasing NLRX1 expression via miR-195**. The injured neurons were introduced with oe-NKILA + sh-NC, oe-NKILA + sh-NLRX1, miR-195 inhibitor + sh-NC, miR-195 inhibitor + sh-NLRX1, sh-NLRX1 or sh-NC. (**A**) cell proliferation of injured neurons after treatment measured by EdU assay. (**B**) quantitative analysis for cell apoptosis of injured neurons after treatment measured by flow cytometry. (**C**) LDH content in injured neurons after treatment. (**D**) protein expression of NLRX1 and apoptosis-related factors (Bcl-2, Bax and Caspase-3) in injured neurons after treatment measured by Western blot analysis. * *p* < 0.05 compared with the treatment of sh-NC. # *p* < 0.05 compared with the treatment of oe-NKILA + sh-NC. & *p* < 0.05 compared with the treatment of miR-195 inhibitor + sh-NC. All data were measurement data and expressed as mean ± standard deviation. Unpaired *t* test was used for comparison between two groups. The one-way ANOVA was used for comparison among multiple groups, followed by Tukey’s post-hoc test. The cell experiment was repeated three times.

In order to further investigate the mechanism of NLRX1 in injured neurons, injured neurons were treated with sh-NLRX1 alone or in the presence of oe-NKILA or miR-195 inhibitor. The results displayed that NLRX1 expression was markedly decreased after the treatment of sh-NLRX1 (Figure 5D). Then, EdU assay and flow cytometry were conducted to measure the neuronal proliferation and apoptosis in injured neurons. As shown in Figure 5A, 5B, the injured neurons showed decreased cell proliferation and increased cell apoptosis following the treatment of sh-NLRX1. The results in Figure 5C demonstrated that silencing NLRX1 led to increased LDH content. Western blot analysis revealed that the expression of Bcl-2 was decreased, while the expression of Bax and Caspase-3 was increased in injured neurons after silencing NLRX1 (Figure 5D). Nonetheless, the treatment of sh-NLRX1 decreased the expression of NLRX1 and Bcl-2, as well as in cell proliferation, but increased Bax and Caspase-3 expression, LDH content and cell apoptosis in the presence of oe-NKILA and miR-195 inhibitor (Figure 5A–5D). Taken together, the upregulation of NKILA promotes functional recovery of injured neurons *via* the upregulation of miR-195-dependent NLRX1.

### Astrocytes transfer NKILA to neurons *via* EVs

**Figure 6:**
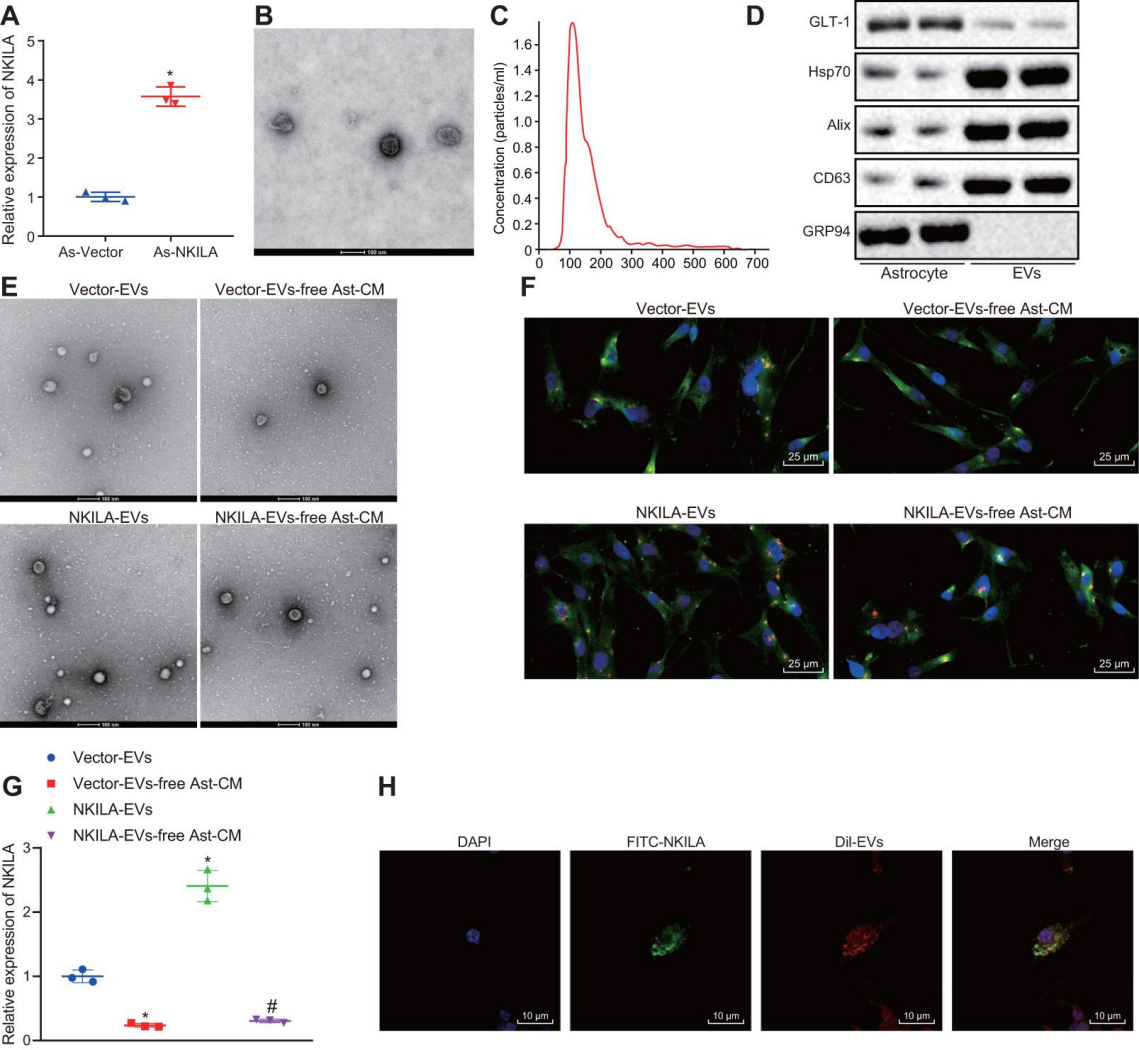
**Astrocytes transfer NKILA into neurons through EVs**. (**A**) NKILA expression in astrocytes detected by RT-qPCR. (**B**) representative electron micrograph of EVs isolated from astrocytes (scale bar: 100 nm). The red arrow points to the EVs. (**C**) size and particle distribution plots of isolated EVs from culture medium by NTA. (**D**) expression of GLT-1, Hsp70, CD63 and Alix as well as the negative marker GRP94 evaluated by Western blot analysis. (**E**) observation of EVs released by astrocytes under an electron microscope (scale bar: 100 nm). (**F**) microscopic views of uptake of EVs by neurons (× 400). (**G**) the expression of NKILA in the supernatant of the astrocyte-derived EVs and EV-free Ast-CM by RT-qPCR; * *p* < 0.05 compared with the Vector-EVs. # *p* < 0.05 compared with the treatment of NKILA-EVs. (**H**) uptake of EVs by neurons observed by the confocal laser microscopy (scale bar: 10 μm). All data were measurement data and expressed as mean ± standard deviation. Unpaired *t* test was used for comparison between two groups. One-way ANOVA, followed by Tukey’s post-hoc test was used for multi-group comparison. The cell experiment was repeated three times.

To validate whether NKILA was delivered from astrocytes to neurons by EVs, NKILA in astrocytes were overexpressed. As depicted in Figure 6A, NKILA expression was significantly increased in astrocytes after NKILA overexpression, validating the successful infection efficiency. The characterization of EVs using TEM and nanoparticle tracking analysis demonstrated that EVs ranged from 50 to 500 nm in diameter in cup-shaped morphology (Figure 6B, 6C). As shown in Figure 6D, Western blot analysis showed positive expression for Hsp70, CD63 and Alix, but negative expression for GRP94, while GLT-1 was highly expressed in astrocytes. It was observed that the uptake of EVs by neurons was notably elevated after overexpression of NKILA (Figure 6E, 6F). We next examined the expression of NKILA in EVs isolated from astrocytes. The results showed that the expression of NKILA was mainly distributed in EVs, and the expression of NKILA in the derived EVs was increased significantly in EVs extracted from astrocytes overexpressing NKILA (Figure 6G). Afterwards, astrocytes were infected with FITC-labeled NKILA-containing EVs. EVs were then extracted and incubated with Dil-labelled-neurons (red) for 48 h. The co-localization of FITC and Dil were observed in the neurons under the confocal laser microscopy (Figure 6H). It was found that FITC-NKILA-containing EVs were internalized in the neurons. Coherently, NKILA was transferred from astrocytes into neurons *via* EVs.

### Astrocyte-derived EVs deliver NKILA to promote the recovery of injured neurons by regulating miR-195-mediated NLRX1 *in vitro*

**Figure 7:**
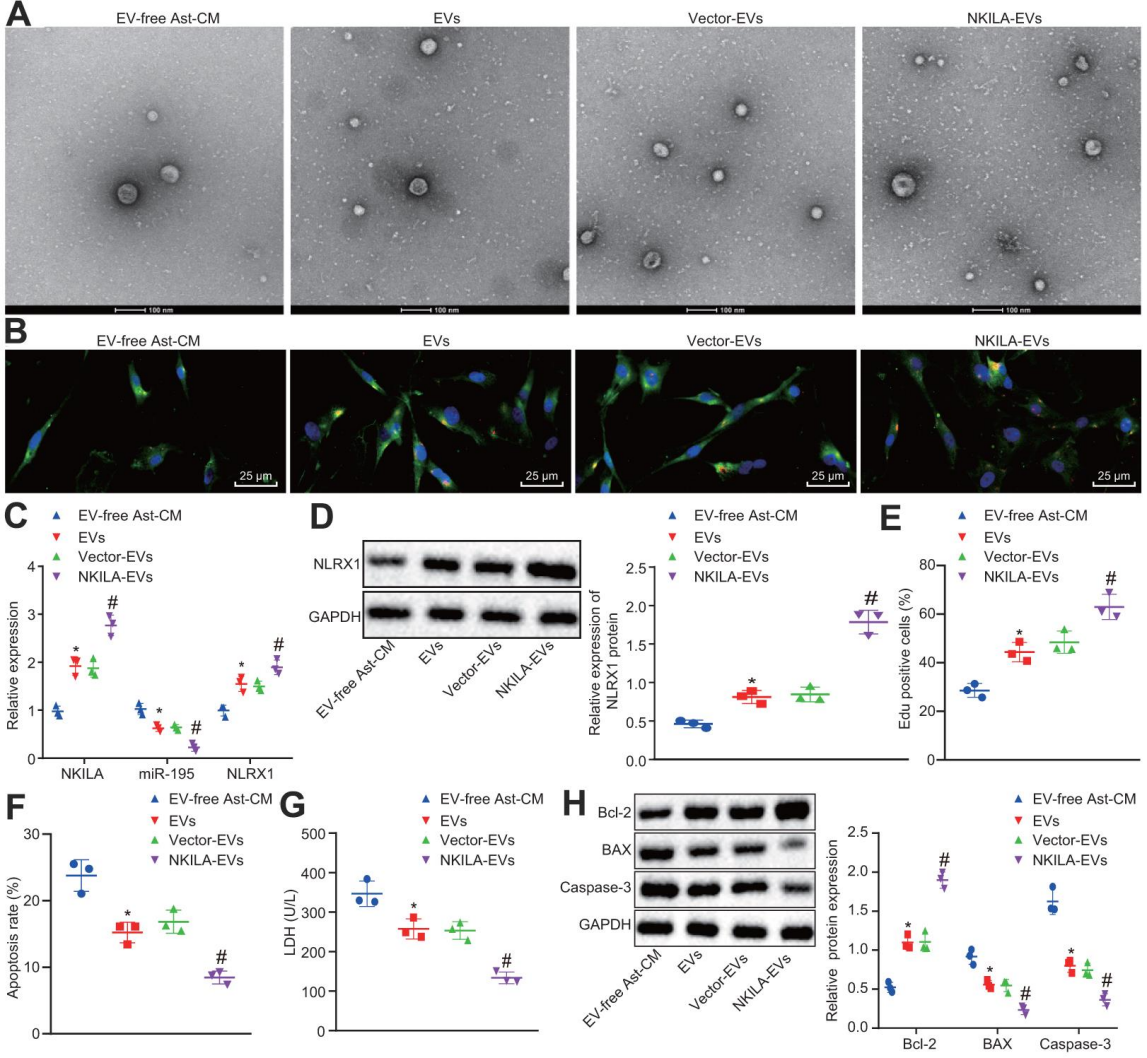
**NKILA delivered by astrocyte-derived EVs promotes neuron recovery by increasing NLRX1 expression via miR-195 *in vitro*.** The injured neurons were treated with NKILA-EVs and vector-EVs. (**A**) observation of EVs released by astrocyte under an electron microscope (scale bar: 100 nm). (**B**) microscopic views of uptake of EVs by neurons (× 400). (**C**) expression of NKILA, miR-195 and NLRX1 in injured neurons measured by RT-qPCR. (**D**) protein expression of NLRX1 in injured neurons measured by Western blot analysis. (**E**) cell proliferation of injured neurons after treatment measured by EdU assay. (**F**) quantitative analysis for cell apoptosis of injured neurons after treatment measured by flow cytometry. (**G**) LDH content in injured neurons after treatment. (**H**) expression of Bcl-2, Bax and Caspase-3 in injured neurons after treatment measured by Western blot analysis. * *p* < 0.05 compared with neurons co-cultured with EV-free Ast-CM. # *p* < 0.05 compared with neurons co-cultured with Vector-EVs. All data were measurement data and expressed as mean ± standard deviation. Unpaired *t* test was used for comparison between two groups. One-way ANOVA, followed by Tukey’s post-hoc test, was used for multi-group comparisons. The cell experiment was repeated three times.

The results by electron microscope showed that compared with neurons co-cultured with EV-free Ast-CM, more EVs were observed in those co-cultured with EVs. There was no significant difference between neurons co-cultured with EVs and vector-EVs. Neurons co-cultured with NKILA-enriched EVs had increased EVs in comparison with those co-culture with vector-EVs (Figure 7A, 7B). In order to classify the specific mechanism of NKILA delivered by astrocyte-derived EVs in injured neurons, RT-qPCR was initially conducted to measure the expression of NKILA, miR-195 and NLRX1 in injured neurons co-cultured with EVs. The results displayed that compared with injured neurons co-cultured with EV-free Ast-CM, those co-cultured with EVs showed elevated NKILA expression and mRNA and protein expression of NLRX1, while reduced miR-195 expression. There was no significant difference in NKILA, NLRX1 and miR-195 expression between neurons co-cultured with EVs or vector-EVs. Relative to vector-EVs, the injured neurons co-cultured NKILA-enriched EVs showed an increased NKILA expression and the mRNA and protein expression of NLRX1, but decreased expression in miR-195 (Figure 7C, 7D). As displayed in Figure 7E–7H, further analysis demonstrated that in comparison with neurons co-cultured with EV-free Ast-CM, those co-cultured with EVs displayed increased proliferation and Bcl-2 expression, while diminished apoptosis, LDH content as well as Bax and Caspase-3 expression. Neurons co-cultured with EVs or vector-EVs exhibited similar capacities in proliferation and apoptosis as well as LDH content. By contrast, co-culture with NKILA-enriched EVs contributed to enhancements in neuronal proliferation and Bcl-2 expression, whereas declines in neuronal apoptosis, LDH content as well as Bax and Caspase-3 expression. These data thus underpin the pivotal role of NKILA cargo of EVs on neurons in injury repairing by downregulating miR-195 to restore NLRX1.

### Astrocyte-derived EVs loaded with NKILA promote brain recovery in TBI mice *in vivo*

**Figure 8:**
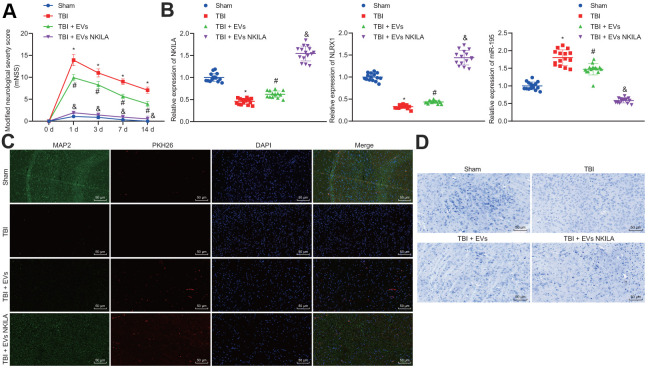
**Astrocyte-derived EVs loaded with NKILA promote brain recovery in TBI mice *in vivo*.** Mice were randomly classified into sham-operated mice, TBI mice, TBI mice treated with EVs or NKILA-enriched EVs (15 mice/group). (**A**) the mNSS determined before TBI and on the 1, 3, 7 and 14 days after TBI. (**B**) the levels of NKILA and NLRX1 in mouse left cerebral cortex tissue determined with RT-qPCR. (**C**) the expression of neuron marker MAP2 in PKH26-labeled EVs assayed with immunofluorescence assay (× 200). (**D**) The loss of neuron cells assessed using Nissl staining (× 200). * *p* < 0.05 compared with sham-operated mice. # *p* < 0.05 TBI mice. & *p* < 0.05 TBI mice treated with EVs. N = 15. All data were measurement data and expressed as mean ± standard deviation. One-way ANOVA was used for comparison among multiple groups, followed by Tukey’s post-hoc test. Comparisons between time-based measurements on neuronal function were determined with repeated measures ANOVA.

To validate the efficacy of astrocyte-derived EVs, NKILA-containing EVs were injected into a mouse model of TBI. Results demonstrated that TBI mice exhibited higher modified nervous system severity score (mNSS) on day 3 and continued on day 14, while marked reduction was observed upon EV treatment on TBI mice, and mice treated with NKILA-enriched EVs exhibited the most markedly lower mNSS (Figure 8A). Thereafter, we isolated mouse left cerebral cortex tissue and RT-qPCR found that compared with the sham-operated mice, the levels of NKILA and NLRX1 in the TBI mice were significantly reduced, while the level of miR-195 increased, while an opposite trend was observed upon EV treatment and mice treated with NKILA-enriched EVs exhibited a more significantly opposite trend compared with TBI mice (Figure 8B). Immunofluorescence was performed to assess the expression of neuron marker MAP2 in PKH26-labeled EVs, revealing that the expression of MAP2 was reduced in TBI mice compared with the sham group, while its expression was increased in response to EV treatment in TBI mice, and significantly higher in TBI mice treated with NKILA-enriched EVs. Meanwhile, the presence of red fluorescence in TBI mice treated with EVs or NKILA-enriched EVs indicated that the EVs successfully transferred the brain tissue (Figure 8C). As expected, the results of Nissl staining supported the results of immunofluorescence (Figure 8D). Therefore, astrocyte-derived EVs loaded with NKILA could promote brain recovery in TBI mice.

## DISCUSSION

TBI is a major public health and socio-economic problem across the world with high mortality rate, culminating in lifelong disability in survived TBI patients [24]. A diversity of lncRNAs and miRNAs has been showed to display altered expression in TBI [25]. Moreover, a study revealed that EVs derived from multipotent mesenchymal stromal cells promotes the functional recovery in TBI rats *via* increased endogenous angiogenesis and neurogenesis, as well as decreased in inflammation [26]. In the present study, we observed that NKILA delivered by astrocyte-derived EVs could suppress neuronal injury and enhance brain recovery following TBI by elevating miR-195-targeted NLRX1.

Initially, the study revealed that NKILA was downregulated in injured neurons, while upregulated NKILA promotes neuronal proliferation but inhibited neuronal apoptosis and LDH content. It has been reported that NKILA was poorly expressed in esophageal squamous cell carcinoma tissues and cells [14], which is consistent with our results. Furthermore, a study conducted by Wang et al. revealed that lncRNAs were aberrantly expressed in rats with TBI [13]. For instance, lncRNA UCA1 expression was revealed to be decreased in epilepsy and seizure-induced brain injury [27], which was in line with our results. Recently, accumulating evidence suggests that NKILA suppressed NF-κB which could lead to cell death and behavioral deficits in mice with TBI [14, 28]. Moreover, a prior study highlighted that lncRNA CasC7 overexpression suppressed neuronal apoptosis in rats with spinal cord ischemia-reperfusion injury [29]. LDH was commonly used as a marker to evaluate neuronal injury [30]. The decreased LDH content is correlated with the neuroprotective effects after TBI in rat [31], which is consistent with our results that overexpression of NKILA induced marked reduction in LDH content.

Besides, NKILA increased NLRX1 expression by competitively binding to miR-195. The NLRX1 expression was decreased in neurons after NKILA inhibition and miR-195 upregulation. A prior study suggested that lncRNAs could competitively binds to miRNAs to regulate the expression of RNA targets [32]. For example, Wu et al. observed that lncRNA PVT1 directly bound to miR-195 and mediated its expression [33]. Another study suggested that miR-195 directly targeted and bound to NLRX1 and then downregulated its expression [19]. Moreover, our results also showed that silencing NLRX1 promotes neuronal injury by increasing neuronal apoptosis and LDH contents, and inhibiting neuronal proliferation. A recent literature has emerged in providing findings that the downregulation of NLRX1 dampens neuronal tissue damages after brain injury [23]. In addition, it was interesting to note that NLRX1 inhibits inflammation and apoptosis, which protects against myocardial ischemic injury [34].

Furthermore, the present study also demonstrated that the co-cultured astrocytes upregulated NKILA in injured neurons, and astrocyte-derived EVs delivers NKILA promoted neuronal proliferation and repressed neuronal apoptosis. As reported, astrocytes could secrete signaling molecules to protect the CNS through interactions with neurons and other glial cells [22]. Interestingly, an emerging study indicated that on the basis of prion protein astrocyte-derived EVs resulted in the improvement of neuronal survival [35]. Furthermore, a study showed consistent results with our study, where the glioma cell-derived EVs increased the long intergenic non-coding RNA CCAT2 (linc-CCAT2) expression in human umbilical vein endothelial cells (HUVECs), and the glioma cell-derived EVs containing linc-CCAT2 enhanced HUVEC angiogenesis and inhibited HUVEC apoptosis [36]. The purpose of this study was to explore the mechanism of EVs in protecting neurons from injury at the cellular level. While *in vivo*, as an important part of CNS, astrocytes are closely adjacent to neurons and may provide nutrients and supporting for neurons [37]. The released EVs serve as a cargo carrying NKILA and phagocytosed by neurons, thus releasing NKILA into neurons and playing its therapeutic role [38]. Also, Frühbeis et al. proposed a novel mode of reciprocal neuron-glia communication with the involvement of neurotransmitter-mediated transfer of EVs from oligodendrocytes to neurons [39], whether this kind of mode functioned in neuronal injury warrant further experiments. The detailed mechanisms of NKILA-induced suppression of neuronal injury *in vivo* were then substantiated in the present study, demonstrating that astrocyte-derived EVs loaded with NKILA promote brain recovery in TBI mice.

**Figure 9:**
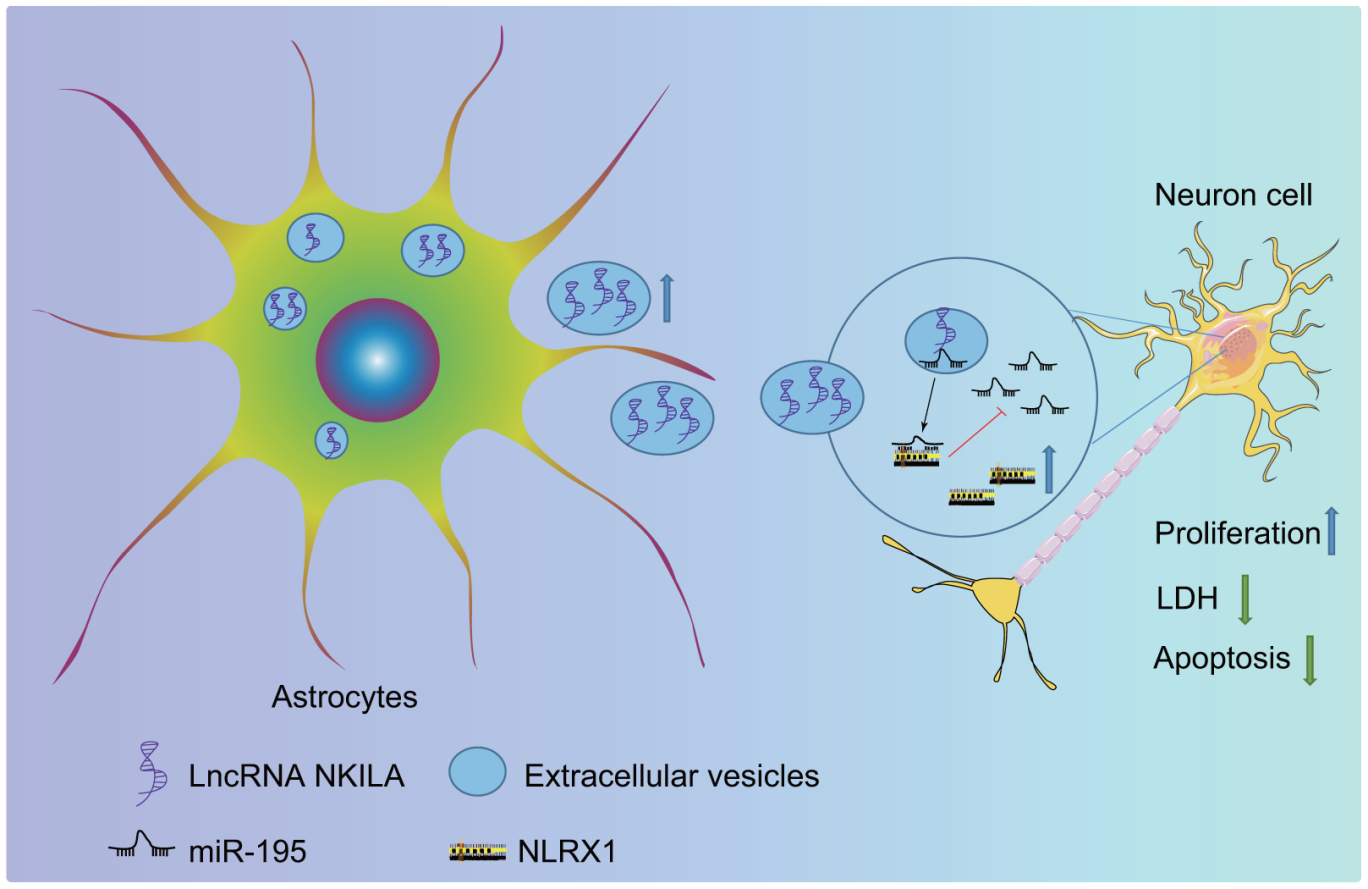
**The molecular mechanism of astrocyte-secreted EVs enriched with NKILA in TBI**. Astrocytes secrete EVs to stimulate NKILA expression in neurons, which upregulates NLRX1 by competitively binding to miR-195 and prevents neuronal injury following TBI.

To sum up, the present study delineates that the upregulation of NKILA in astrocyte-derived EVs upregulates NLRX1 by inhibiting miR-195, therefore exerting suppressive effect on neuronal injury in TBI (Figure 9). Thus, astrocyte-derived EVs enriched with NKILA may serve as a promising new target for the development of therapeutic strategies for TBI.

## MATERIALS AND METHODS

### Ethics statement

The animal protocols were approved by the Ethics Committee of Shanghai Pudong New Area People’s Hospital. All efforts were made to minimize the suffering of animals.

### Cell culture

Human brain astrocytes (Catalog #1800, ScienCell, Carlsbad, CA, USA) were cultured in astrocyte medium (Catalog Number: 1801) and human brain neurons (Catalog #1520, ScienCell, Carlsbad, CA, USA) in neuronal medium (Catalog Number: 1521). The cells were grown in a humidified atmosphere of 5% CO_2_ at 37° C. The culture medium was renewed twice every week. Upon 90% cell confluence, cells were counted with a Countess Automated Cell Counter (Invitrogen Inc., Carlsbad, CA, USA). The astrocytes were cultured in Dulbecco’s modified Eagle’s medium (DMEM, Lonza, Morristown, NJ, USA) supplemented with 10% heat-inactivated fetal bovine serum (FBS), 1% L-glutamine and 1% penicillin/streptomycin/amphotericin B. The expression of neuronal markers MAP2 and NeuN were evaluated with immunofluorescence assay.

### Development of neuronal injury model *in vitro*

Human brain neurons were incubated *in vitro* for 7 days, and then the medium was renewed with serum-free DMEM. After 24 h, neuronal injury experiments were conducted as performed in prior studies [40, 41]. Briefly, the plastic dropper of a micropipette was used to mechanically cut incubated neurons in culture wells. The cut neurons were graded based on mild cutting, moderate or severe degree of cutting. The neurons received the same treatment as the injured neurons except mechanical cutting served as control. The cell proliferation and LDH content in culture supernatant after injury were detected using the cell counting kit (CCK)-8 assay and an LDH kit respectively at different time points (30 min, 1 h, 6 h, 12 h, 24 h), to evaluate the degree of injury.

### Cell treatment

Neurons were cultured in a 6-well plate at the density of 2 × 10^5^ cells/well. When the cell confluency reached 80%, the neurons were transfected with plasmids expressing oe-NKILA, sh-NKILA or sh-NLRX1, miR-195 inhibitor, or miR-195 mimic as per the instructions of lipofectamine 2000 (11668-019, Invitrogen, New York, CA, USA). The cell transfection was conducted by Shanghai GenePharma Co., Ltd. (Shanghai, China). After transfection, the neurons were incubated with 5% CO_2_ at 37° C for the following experiments.

### Co-culture of astrocytes and injured neurons

Neuron suspension was seeded into the 6-well plate at the density of 1 mL/well using Transwell system (Corning Inc., Corning, NY, USA). Then, each well was seeded with 1.5 mL astrocyte suspension into the Transwell system and co-cultured for 48 h.

### Construction of lentiviral vectors

The Platinum Pfx DNA Polymerase (Invitrogen, Carlsbad, CA, USA) was used to amplify the cDNA coding NKILA which was then sub-cloned into the Kpn I and BamH I sites of the pcDNA3.1-GFP-puro vector (Invitrogen, Carlsbad, CA, USA). The primer sequences of overexpressed NKILA were as follows: 5'-GGGGTACCAGACCCGGCACCCGCGCAA-3' (sense) and 5'-CGGGATCCCCAGTTAAATTGAGATATACTTACAC-3' (antisense). After that, HEK-293 cells were transfected with the all above-described packaging plasmids. The lentiviral supernatants were collected at day-3, filtered and added to the astrocytes (1 × 10^5^ cells/mL). RT-qPCR was performed to determine the expression of NKILA in the obtained stable cells. Control astrocytes were transfected with the lentiviral pcDNA3.1-YFP-puro vector control (“vector”).

### Isolation and identification of EVs from astrocytes

The culture medium was centrifuged at 110,000 ×g for 18 h at 4° C to prepare EV-free culture medium (EV-free CM) [42]. Prior to the EV extraction, the astrocytes were washed 2 times with phosphate buffer saline (PBS) and cultivated in EV-free culture medium for 48-72 h with the supernatant collected. The EV extraction was performed by differential centrifugation at 4° C. The cell debris and dead cells were removed by centrifugations at 1,200 r/min for 10 min and at 3,200 r/min for 20 min in a refrigerated centrifuge (Eppendorf, Hamburg, Germany). The supernatant was centrifuged at 15,000 r/min for 45 min with a high-speed centrifuge (Beckman Coulter Life Sciences, Brea, CA, USA) to eliminate cell debris and large vesicles and filtered through a 0.22 μm membrane. The filtered medium was then centrifuged by the high-speed centrifuge (Beckman Coulter Life Sciences, Brea, CA, USA) at 30,000 r/min for 90 min to extract EVs. The supernatant was collected as astrocyte culture medium free of EVs (EV-free Ast-CM), while the precipitation was resuspended with PBS and centrifuged again. Afterwards, the precipitation was resuspended with 50-100 μL PBS and then stored at -80° C.

The purity of EVs was identified by a transmission electron microscope (TEM), NanoSight’s nanoparticle tracking analysis (NTA) and Western blot analysis. The EVs were resuspended with 4% paraformaldehyde. The 5 μL suspension was dropped on the electron microscope grid, and fixed for 20 min. The samples were incubated with 1% glutaraldehyde for 5 min, uranyl oxalic acid solution (pH = 7) for 5 min, and hydroxypropyl methyl cellulose on ice for 10 min. After removal of the excess liquids, the samples were allowed to stand for 5-10 min and observed under a TEM. A total of 20 μg EVs was dissolved in 1 mL PBS and vortexed for 1 min to maintain the uniform distribution of EVs. The EV suspension was slowly injected into the one-time clean sample pools to avoid bubble formation. The sample pools were covered with shells and analyzed using a TEM. NTA post-acquisition settings remained unchanged for all samples, and each video was analyzed to calculate the median vesicle size and concentration estimates. The detection was conducted in accordance with the instrument operation specifications. EV size distribution and concentration were analyzed using a NanoSight LM10 system equipped with a fast video capture and particle-tracking software. EVs were visualized by dynamic light scattering with an NTA software that could track Brownian motion of individual EVs. The concentration of EV protein was determined by bicinchoninic acid assay and Western blot analysis was performed to validate the expression of EV-specific surface markers (Hsp70, CD63 and Alix) as well as the endoplasmic reticulum marker GRP94 and astrocyte marker GLT-1 [43, 44].

### Labeling and transferring of astrocyte-derived EVs

The lentivirus-infected astrocytes were labeled using FITC-NKILA (green) to obtain EVs. Next, 20 μg EVs were dissolved in 1 mL of PBS to prepare an EV suspension. Dil staining solution was diluted with EV suspension at the ratio of 1: 1000 and placed at 4° C for 15 min. The mixture was washed with PBS (100,000 g, 70 min). The neurons were fixed with 4% paraformaldehyde, and the nuclei were stained with 4′6-diamidino-2-phenylindole (DAPI) in blue. Dil-traced EVs were co-cultured with neurons for 24 h, and the uptake of EVs by neurons was observed under a laser confocal microscopy. To investigate the recovery effect of astrocyte-derived EVs containing NKILA on injured neurons, the injured neurons were co-cultured with EV-free Ast-CM (CM in the absence of EVs), EVs (neurons + EVs derived from astrocyte), EVs derived from vector astrocyte (neurons + EVs derived from astrocyte treated with lncRNA NKILA-vector) or EVs derived from NKILA-overexpressed astrocyte (neurons + EVs derived from astrocyte treated with oe-NKILA).

### Uptake of astrocyte-derived EVs

EVs were labeled using PKH26 Red Fluorescent Cell Linker Kit. According to the reagent instructions, 1 mL EVs were added into 1 mL PKH26 Red Fluorescent Cell Linker Kit for 3 min, following addition of an equal volume of serum for 1 min to terminate the reaction. The termination reaction solution was diluted with an equal volume of serum medium. After washed with serum-free medium for 3 times, the precipitate was resuspended with 200 μL PBS. The labeled EVs at the original concentration of 5 μL was added into 24-well plates containing cell slides at different time points and incubated for 8 h. After washed with PBS, the cell slides at different time intervals were fixed with 4% paraformaldehyde for 15 min. Nucleus were stained with 10% DAPI for 10 min. Cell slides were sealed with an anti-fluorescent quencher. Images were captured by a confocal microscope and analyzed by Image J software.

### Bioinformatics analysis for target genes of miRNA

The target genes of miR-195 were predicted using the mirDIP database (http://ophid.utoronto.ca/mirDIP/index.jsp#r), where the target genes with Integrated Score > 0.6 were selected. The target genes ranked the top 1000 in the starBase database (http://ophid.utoronto.ca/mirDIP/index.jsp#r) and ranked the top 100 in the miRSearch database (https://mirmap.ezlab.org/) were selected as well. Also, the target genes with miRmap score > 80 were selected in the miRmap (https://mirmap.ezlab.org/). Finally, the results of all databases were intersected using jvenn (http://jvenn.toulouse.inra.fr/app/example.html).

### Measurement of LDH

When the cell membrane was injured after cell necrosis, intracellular enzyme LDH leaked into culture medium. The supernatants of neurons with different treatments were plated into a 96-well plate at 50 μL per well, with 3 duplicated wells with each treatment. LDH contents were measured according to the instructions of an LDH kit (Takara, Tokyo, Japan).

### 5-ethynyl-2'-deoxyuridine (EdU) assay

The neurons at logarithmic growth phase were seeded into a 96-well plate with 200 μL per well. The cells in each well were incubated with EdU culture medium for 1 d and fixed with 50 μL PBS containing 4% polyformaldehyde for 30 min. Then cells were incubated with 50 μL 2 mg/mL decolorizer glycine for 5 min and 100 μL PBS containing 0.5% TritonX-100 for 10 min. After that, cells in each well were incubated with 100 μL 1 × Apollo dyeing in the dark at room temperature for 30 min and washed 3 times with 100 μL PBS containing 0.5% TritonX-100. After DNA staining, each well was incubated with 100 μL DAPI in the dark at room temperature for 30 min, and then washed 3 times with 100 μL PBS. Finally, image acquisition and analysis were carried out.

### Flow cytometry

Apoptosis was detected using Annexin V-FITC/PI detection kits (Nanjing KeyGen Biotech Co., Ltd., Nanjing, Jiangsu, China). After 48-h of culture, neurons were collected with 0.25% ethylene diamine tetraacetic acid-free trypsin, washed 2 times with ice-cold PBS, and suspended in 500 μL binding buffer. The cells were then incubated with 5 μL Annexin V-FITC-labeled specific antibody and PI in the dark for 15-20 min, followed by detection in a flow cytometer and analyzed using the FASCDiva 4.1 software (BD Biosciences, Franklin Lakes, NJ, USA). The cells labeled with Annexin V were regarded early apoptotic cells, while cells labeled with Annexin V and PI were regarded as late apoptotic cells. Single positive cells labeled with PI were necrotic cells, while live cells were labeled with neither. The early and late apoptotic cells were counted.

### FISH assay

The sub-localization of NKILA in neurons was identified using FISH according to the instructions of Ribo^TM^ lncRNA FISH Probe Mix (Red; Guangzhou Ribo Biology Co., Ltd., Guangzhou, Guangdong, China). The neurons were seeded into the plate at 6 × 10^4^ cells/well and cultured until the cell confluency reached about 80%. Then, the neurons were fixed by 1 mL 4% paraformaldehyde, treated with proteinase K (2 μg/mL; Sigma-Aldrich Chemical Company, St Louis, MO, USA), glycine (YZ-140689; Beijing solarbio science and technology Co., Ltd., Beijing, China) and acetamidine reagent. The slides were then incubated with 250 μL pre-hybridization solution at 42° C for 1 h and with 250 μL 300 ng/mL hybridization solution containing NKILA probe overnight at 42° C. After that, the slides were stained for 5 min with DAPI (ab104139, 1: 100, Abcam Inc., Cambridge, UK) diluted using PBS-Tween 20. The slides were then mounted with an anti-fluorescent quencher. Five different fields were selected under a fluorescence microscope (Olympus Optical Co., Ltd, Tokyo, Japan) for observation and photographs.

### Dual-luciferase reporter gene assay

The sequences of miR-195 and NLRX1 3’-untranslated region (3’UTR) were artificially synthesized, and the gene promoter fragments were introduced into ARE-Luc reporter gene (Yeasen Company, Shanghai, China) using endonuclease sites Nhe I and Bgl II. The mutation sites in complementary sequence of seed sequences were designed on the WT of miR-195 and NLRX1. After restriction endonuclease digestion, the target fragments were inserted into the pGL3 reporter plasmids using T4 DNA ligase to obtain luciferase reporter plasmid, such as pGL3-miR-195-WT, pGL3-NLRX1-WT, pGL3-miR-195-MUT, pGL3-NLRX1-MUT and pGL3-control plasmids. The above plasmids with correct sequencing were co-transfected with NKILA overexpression, NKILA NC, miR-195 mimic and miR-195 NC into HEK293T cells (American Type Culture Collection, Manassas, VA, US; http://www.bnbio.com/pub/search.asp?Keyword=HEK-293T). After 12-h of transfection, the cells were treated with dimethyl sulfoxide (DMSO) for 24 h, and lysed with buffer following protocols of a Dual-Luciferase Reporter Assay kit (E1910, Promega Corporation, Madison, WI, USA). Luciferase activity was measured using a Glomax20/20 luminometer fluorescence detector (Promega, Madison, WI, USA).

### RNA pull-down assay

Neurons were treated with 50 nM biotinylated NKILA-WT (WT-Bio-NKILA) and NKILA-MUT (MUT-Bio-NKILA), respectively. After 48 h, the cells were lysed with specific lysis buffer (Ambion, Austin, TX, USA) for 10 min. The lysate was then incubated with M-280 streptavidin magnetic beads (S3762, Sigma-Aldrich, St. Louis, MO, USA) pre-coated with RNase-free bovine serum albumin (BSA) and yeast tRNA (TRNABAK-RO, Sigma-Aldrich, St Louis, MO, USA) at 4° C for 3 h. The beads were then rinsed 2 times with pre-cold lysis buffer, 3 times with low-salt buffer and one time with high-salt buffer respectively. The combined RNA was then purified using the Trizol reagent, followed by RT-qPCR to analyze the enrichment of miR-195.

### RNA immunoprecipitation (RIP) assay

The binding of NKILA and miR-195 with Argonaute 2 (AGO2) was detected using a Magna RIP RNA-Binding Pretein Immunoprecipitation kit (Millipore Inc., Bedford, MA, USA). The neurons were lysed with equal volume of radio-immunoprecipitation assay (RIPA) lysis buffer (P0013B, Beyotime Biotechnology Co., Shanghai, China) on ice for 5 min, and then centrifuged at 14,000 rpm for 10 min at 4° C. Part of the cell extract was used as Input, while the remaining was probed with antibody for co-precipitation. The cell exact was incubated with rabbit polyclonal antibodies against IgG (ab109489, 1: 100, Abcam, Cambridge, UK; used as NC) or AGO2 (ab32381, 1: 50, Abcam, Cambridge, UK) for co-precipitation. In details, 50 μL magnetic beads of each system were resuspended with 100 μL RIP wash buffer (EHJ-BVIS08102, Xiamen Jiahui Biotechnology Co., Ltd., Xiamen, Fujian, China), followed by an incubation with 5 μg antibody according to experimental groups. After washing, the magnetic bead-antibody complex was resuspended in 900 μL RIP Wash Buffer and incubated with 100 μL cell exact at 4° C overnight. The samples were placed on the magnetic pedestals to collect bead-protein complexes. After detachment using proteinase K buffer, RNA was extracted for RT-qPCR analysis.

### Animal model of TBI

All experimental procedures were performed in accordance with the current regulations of the laboratory animal care and use guidelines. Sixty C57BL/10ScNJ male adult mice aged 10-12 weeks, weighed 20-22 g were obtained from SJA Laboratory Animal Co., Ltd (Hunan, China). Mice were randomly classified 4 groups, sham-operated mice, TBI mice, TBI mice treated with EVs or NKILA-enriched EVs (15 mice/group). All animals were maintained in a controlled environment under a 12-h light/dark cycle at 22 ± 3° C and were offered with standard rodent nutrition and water. The mouse model of TBI was induced as a prior study [45]. Mice were anesthetized with 1% sodium pentobarbital (50 mg/kg body weight) administered intraperitoneally (ip), and then the TBI 0310, a pneumatic impacting device (Precision Systems and Instrumentation, Fairfax Station, VA) with a hard stop Bimba cylinder (Bimba Manufacturing, Monee, IL) was adopted to perform a unilateral, moderately controlled cortical impact (CCI) of 2.0 mm depth at 3.5 m/ s and 500 ms dwell time on mice. The size of the bevelled impactor was 5 mm. All craniotomies were put midway between the bregma and lambda sutures in the left hemisphere of the brain. As for the sham-operated mice, only the bone window was opened.

### Stereotactic injection of EVs into mouse brain

Mice were deeply anesthetized with 1.2% Avertin (Sigma; #T48402) prior to EV injection. EVs were fluorescently stained with the lipophilic dye PKH26 (Sigma Aldrich) as per the manufacturer's protocol. Wild-type C57 mice received direct microinjections by stereotactic placement into the injured brain area (motor cortex, AP:1.0 mm, ML: 0.7 mm, DV: 1.2 mm). Next, 2 μL saline containing EVs with NKILA (~ 3 ± 0.75 μg protein) was injected into the injured brain area at the speed of 100 nl/min (Narishige Scientific Instrument lab, Japan) [46]. Following neurobehavioral assessment analysis, the mice were euthanized and separated, after which the left cerebral cortex tissues were collected for following research.

### Neurobehavioral assessment

The mNSS test was adopted to evaluate neurological function. The test was performed before TBI and on the 1, 3, 7 and 14 days after TBI. The scale ranges from 0 to 18 points (normal score, 0 points; maximum deficit score, 18). mNSS consists of movement (muscle state and abnormal movement), sensation (vision, touch and proprioception), reflex response and balance tests. If the mouse fails to complete the test or does not respond as expected, 1 point is score; therefore, the higher the score, the more severe the injury.

### Immunofluorescence assay

Cells or tissues were incubated with anti-MAP2 and NeuN (Abcam) overnight at 4° C, and then incubated with conjugated secondary antibodies for 1 h at room temperature in the dark. After washing several times with PBS, cells were incubated with DAPI for 3 minutes, and then packed in glycerol. A fluorescence microscope (Olympus, Tokyo, Japan) was adopted to observe the immunofluorescence signal.

### Nissl staining

Slices from the anterior and posterior sections were mounted onto slides and stained with 0.2% thionine.

### RT-qPCR

The total RNA of cells and tissues was extracted according to the instructions of the Trizol kit (15596-018, Beijing Solarbio Science and Technology Co., Ltd., Beijing, China). The primers (Table 1) were synthesized by TaKaRa Biotechnology Co., Ltd. (Dalian, Liaoning, China). The reverse transcription was conducted according to the instructions of a miRNA reverse transcription kit (D1801, Ha’erbin Haigene Detection Co., Ltd., Ha’erbin, Leilongjiang, China) and a cDNA reverse transcription kit (K1622, Beijing Reanta Biotechnology Co., Ltd., Beijing, China). Then, RT-qPCR was performed using the fluorescence quantitative PCR instrument (ViiA7, Sun Yat-sen University, DAAN Gene Co., Ltd., Guangzhou, Guangdong, China). With U6 and glyceraldehyde-3-phosphate dehydrogenase (GAPDH) as the internal references, the relative expression of the target gene was analyzed by 2^-ΔΔCt^ method [38].

**Table 1:** Primer sequences of related genes for RT-qPCR.

**Genes**	**Primer sequences (5'-3')**	
LncRNA NKILA	F: CTGGAGAAGTCAAGCCCAGG	R: GTCAGTGCCTCTGAACCTCC
miR-195	F: GGGGTAGCAGCAGCACAGAAAT	R: TCCAGTGCGTGTCGTGGA
NLRX1	F: GAGCTCCGGATTCAGTCGAG	R: CGAGTTTCCATGGTGACGGA
U6	F: GCTTCGGCACATATACTAAAAT	R: CGCTTCACGAATTTGCGTGTCAT
GAPDH	F: CTGACATGCCGCCTGGAGA	R:ATGTAGGCCATGAGGTCCAC

Note: RT-qPCR, reverse transcription quantitative polymerase chain reaction; miR-195, microRNA-195; NLRX1, Nod-like receptor X1; GAPDH, glyceraldehyde-3-phosphate dehydrogenase; F, forward; R, reverse; LncRNA NKILA, long non-coding RNA nuclear transcription factor NF-κB interacting lncRNA.

### Western blot analysis

The total protein of cells and tissues were extracted using high efficiency RIPA lysis buffer (R0010, Beijing Solarbio Science and Technology Co. Ltd., Beijing, China). The samples were centrifuged at 15,000 rpm/min for 1 min. Then, the supernatant was collected to measure the protein concentration using a bicinchoninic acid kit (20201ES76, Yeasen Company, Shanghai, China). The protein was separated with sodium dodecyl sulfate-polyacrylamide gel electrophoresis, and transferred onto a polyvinylidene fluoride (PVDF) membrane using wet transfer method. The membrane was blocked with 5% BSA at room temperature for 1 h, and incubated with primary rabbit polyclonal antibodies to NLRX1 (ab105412, 1: 1,000), Bcl-2-Associated X (Bax; ab53154, 1: 1,000), B-cell lymphoma-2 (Bcl-2; ab196495, 1: 1,000), Cleaved-caspase-3 (ab2302, 1: 500) and GAPDH (ab181602, 1: 10,000) at 4° C overnight. Afterwards, the membrane was incubated with horseradish peroxidase-labeled goat anti-rabbit antibody to IgG (ab205718, 1: 20,000) at room temperature for 1 h. All above antibodies were purchased from Abcam Inc. (Cambridge, UK). Signals were detected by chemiluminescence and densitometry was performed utilizing ImageJ 1.48u software (National Institutes of Health, Bethesda, Maryland, USA). The relative expression was calculated with the ratio of gray value of protein bands to that of GAPDH.

### Statistical analysis

Statistical analysis was performed using GraphPad Prism 6.0 (GraphPad Software, La Jolla, CA, USA). All experiments were repeated 3 times. All data conformed to normal distribution and homogeneity test of variance. The measurement data were expressed as mean ± standard deviation. Comparisons between two groups were analyzed using unpaired *t*-test, while comparisons among multiple groups were tested by one-way analysis of variance (ANOVA) followed by Tukey’s post hoc test for multiple pairwise examinations. Comparisons between time-based measurements on cell viability within each group were performed with two-way ANOVA, followed by Bonferroni's post hoc test. Whereas, comparisons between time-based measurements on neuronal function were determined with repeated measures ANOVA. *P* < 0.05 was considered to be statistically significant.

### Availability of data and material

The datasets generated/analysed during the current study are available.
